# Morphological characteristics and optimized protocols for *in vitro* germination and viability testing of *Idesia polycarpa* Maxim. Pollen

**DOI:** 10.1371/journal.pone.0355205

**Published:** 2026-08-03

**Authors:** Zhoujun Zhu, Bin Xu, Junru Zhao, Li Wang, Yeye Zhang, Ran Yang, Rui Wang, Jugang Wang, Jianxin Li, Chao Gao

**Affiliations:** 1 College of Agro-forestry Engineering & Planning, Tongren University, Tongren, China; 2 Guizhou Provincial Key Laboratory for Biodiversity Conservation and Utilization in the Fanjing Mountain Region, Tongren University, Tongren, China; 3 Guizhou Provincial Education Department Engineered Research Center for Innovation and Utilization of Oil Camelia Germplasm Resources, Tongren University, Tongren, China; 4 Institute for Forest Resources and Environment of Guizhou, Guizhou University, Guiyang, China; Institut National de la Recherche Agronomique, MOROCCO

## Abstract

*Idesia polycarpa* Maxim. is a premier woody oil species in Guizhou Province, China, whose fruit yield and oil quality largely depend on effective pollination and fertilization. However, limited research on pollen viability and germination has hindered industrial progress. To address this gap, a comprehensive evaluation framework for elite *I. polycarpa* germplasm was developed, integrating micromorphological analysis, optimized staining protocols, and *in vitro* germination assay. Scanning electron microscopy (SEM) revealed that *I. polycarpa* pollen, while genetically conserved at the genus level—characterized by prolate shapes, tricolporate apertures, and reticulate exine ornamentation—exhibits notable micromorphological variation among genotypes. Of the nine staining protocols tested (2,3,5-triphenyl tetrazolium chloride [TTC], carbol fuchsin, acetocarmine, methylene blue, Alexander, peroxidase, 2,5-diphenylmonotetrazolium bromide [MTT], I_2_-KI, and red ink), TTC and red ink were the most effective, offering clear chromatic distinction between viable and non-viable pollen. Through orthogonal experimental designs, genotype-specific optimal media for *in vitro* germination were identified: 0.40 g/L H_3_BO_3_, 0.01 g/L KNO_3_, 0.02 g/L Ca(NO_3_)_2_·4H_2_O, and 0.20 g/L KH_2_PO_4_ for STZ-6; and 0.20 g/L H_3_BO_3_, 0.02 g/L KNO_3_, 0.02 g/L Ca(NO_3_)_2_·4H_2_O, and 0.10 g/L KH_2_PO_4_ for STZ-9. Regression analysis confirmed a highly significant positive correlation (*P* < 0.01) between *in vitro* germination rates and the staining results from both TTC and red ink across various concentrations. Notably, 5% TTC and 30% red ink exhibited the highest coefficients of determination. A hierarchical evaluation strategy is thus proposed: the 5% TTC method is recommended for precise laboratory quantification due to its stability, while the 30% red ink method, due to its ease of use, is suited for rapid field-based screening. This study provides valuable insights into the morphological characteristics of *I. polycarpa* pollen and establishes a standardized evaluation framework, supporting germplasm innovation and optimizing pollination management.

## Introduction

*Idesia polycarpa*, a deciduous tree belonging to the genus *Idesia* (Salicaceae), is native to East Asia [1]. In China, it is predominantly found in provinces south of the Qinling Mountains and within the Huaihe River Basin. Its oil yield per unit area can range from 2.25 to 3.75 t·hm^2^ [2]. The oil produced is notable for its high linoleic acid content, which accounts for 66%–81% of the total fatty acids, and is rich in various bioactive compounds [3]. Consequently, it is acclaimed as a “tree-based oil reservoir” and plays a pivotal role in China’s rural revitalization strategy [4]. Guizhou Province, with its ideal climate for *I. polycarpa*, currently maintains an artificial cultivation area of approximately 46,700 hm^2^ [5]. By 2030, the province is projected to develop over 5 million mu of industrial bases, targeting an annual oil production exceeding 200,000 tonnes. However, the expansion of the industry is constrained by the species’ biological traits. As a dioecious plant, fruit yield formation in *I. polycarpa* is heavily reliant on successful pollination and fertilization. Pollen viability, encompassing both fertilization and germination capacity, is a key determinant of reproductive success. Therefore, investigating the morphological features and viability of *I. polycarpa* pollen is of significant theoretical and practical importance for achieving stable yields and boosting industry income.

However, there is currently no research on the screening of pollen viability detection methods or the development of evaluation systems for *I. polycarpa*. Given the significant interspecific differences in pollen characteristics, directly adopting methods from other species may introduce substantial bias [6], which severely hinders efficient artificial pollination, germplasm screening, and breeding programs for this emerging woody oil species. This lack of a standardized assessment framework represents a critical knowledge gap that limits both reproductive biology research and large-scale cultivation management. Therefore, there is a pressing need to develop an efficient pollen viability determination system tailored specifically to *I. polycarpa*.

Studies have shown that characteristics such as pollen size, exine ornamentation, and aperture number have a substantial impact on germination. For example, pollen volume is typically positively correlated with nutrient storage and pollen tube growth rate [7]; exine ornamentation plays a protective role, preserving the structural integrity of the pollen [8]; and the number of apertures determines potential germination sites, contributing to shorter germination times [9]. Thus, understanding the pollen morphological traits of *I. polycarpa* provides the foundational knowledge for exploring its physiological potential. Despite this, current research on *I. polycarpa* has predominantly focused on fruit quality [10,11] and gender expression [12,13], while comprehensive studies on its pollen micromorphology remain scarce. Furthermore, morphological traits alone cannot directly predict pollen performance during germination, making them suitable only as references for assessing pollen viability [14].

In plant breeding and production, the accurate and rapid determination of pollen viability is crucial [15]. While various indirect detection techniques, such as staining methods, are available, they are often limited by their inability to distinguish aborted pollen or their tendency to produce false positives, leading to an overestimation of pollen quality [16]. In contrast, although *in vitro* germination assays are time-consuming and relatively cumbersome, they are considered the most reliable indicator of pollen viability because they directly reflect pollen tube growth and fertilization potential [17,18]. However, *in vitro* germination is highly sensitive to culture conditions [19]. Research has shown that *in vitro* germination is not only influenced by environmental factors such as temperature and humidity but is also significantly affected by medium composition. The concentration ratios and interactions of medium components can markedly alter germination outcomes, with strong interspecific variation and considerable differences in optimal culture systems across species [20]. In field pollination practices or germplasm resource screening, a more efficient and immediate method of viability assessment is often required. To address this, previous studies have focused on identifying staining methods that closely align with *in vitro* germination rates. For instance, red ink and TTC staining have been shown to effectively differentiate between viable and non-viable pollen in *Exochorda racemosa* [21]; in *Lilium longiflorum* [22], acetocarmine staining (49.66%) was found to most accurately reflect actual viability when compared to TTC and I_2_-KI. Similarly, red ink staining was identified as the most suitable method for rapid detection in *Epimedium sagittatum* due to its accuracy and simplicity [23]. Additionally, studies have confirmed a significant linear correlation between 0.3% MTT staining rates and *in vitro* germination rates in *Castanea mollissima* and *C. henryi* (*P* < 0.01), successfully establishing a rapid evaluation system for these species [24]. However, there is currently no research on the screening of pollen viability detection methods or the development of evaluation systems for *I. polycarpa*. Given the interspecific differences in pollen characteristics, directly adopting methods from other species may introduce bias [6]. Therefore, there is a pressing need to develop an efficient pollen viability determination system tailored to *I. polycarpa*.

In this study, pollen from elite *I. polycarpa* individuals was used as research material. SEM was employed to observe pollen morphology, and the effectiveness of nine common staining methods (TTC, carbol fuchsin, acetocarmine, methylene blue, Alexander, peroxidase, MTT, I_2_-KI, and red ink) was systematically evaluated to identify the optimal staining agent. Through single-factor and orthogonal experiments, an efficient *in vitro* germination system was optimized and established as the benchmark for viability evaluation. By analyzing the correlation between staining estimates and *in vitro* germination rates, this work aims to establish a rapid and accurate evaluation system for *I. polycarpa* pollen viability. These findings supplement the limited palynological data for this species, providing a practical reference for selecting suitable male pollinator trees and ensuring efficient artificial pollination in orchard management.

## Materials and methods

### Plant materials

**Fig 1:**
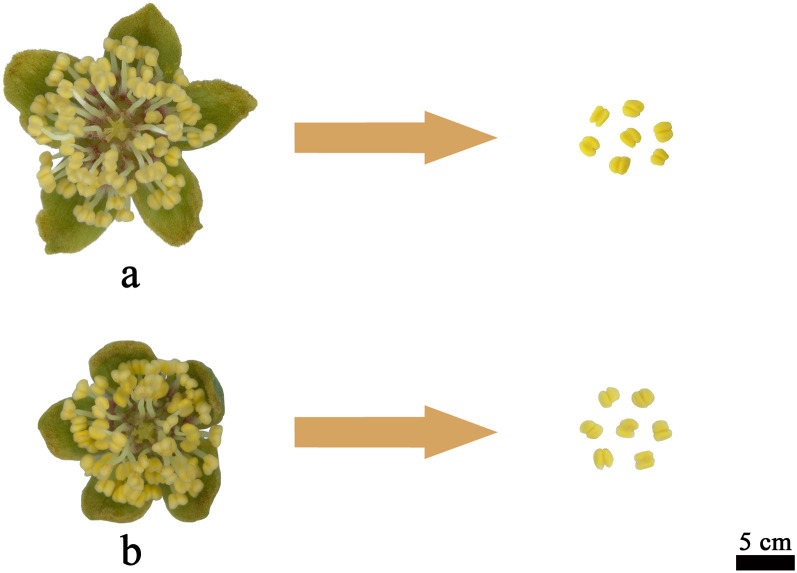
*I. polycarpa* pollen. (a) and (b) represent STZ-6 and STZ-9, respectively.

The sampling site is located at the *I. polycarpa* cultivation base in Guiding County, Guizhou Province (107°25′03″E, 26°59′01″N), characterized by a subtropical humid monsoon climate. The area experiences an annual average temperature of 15.5 °C, a cumulative annual sunshine duration of 1,073.9 hours, a frost-free period of 289 days, annual average precipitation of 1,084.8 mm, and an average relative humidity of 78%. The plantation follows a planting design with a female-to-male ratio of 9:1. For this study, two elite male individuals (pollinizers), designated as ‘STZ-6’ and ‘STZ-9’ (Fig 1), were selected based on the following criteria: (1) high synchronization of flowering phenology with the main female cultivars, (2) long inflorescences, and (3) a large quantity of pollen produced per plant. In early June 2025, branches bearing mature flower buds (just prior to anthesis) were collected from the middle and upper parts of the tree crowns of the selected male trees. These branches were subjected to hydroponic culture at room temperature (15°C). Upon flowering, the flowers were harvested for de-anthering. The collected anthers were dried using a self-made incandescent lamp heating apparatus, maintained at a constant temperature of 30°C for 6 h. Once fully dried, the pollen was promptly sieved and stored in a −80°C ultra-low temperature freezer for subsequent experimental use.

## Experimental methods

### Determination of pollen morphology

Micrographs were captured at magnifications of 500× for the group view, 5,000× for the polar and equatorial views, and 10,000× to resolve the detailed exine ornamentation. The exine ornamentation and aperture shape of the pollen grains were qualitatively characterized based on these microscopic observations. For morphometric evaluation, 25 well-positioned pollen grains were randomly selected across three independent biological replicates to measure the polar axis length (P), equatorial diameter (E), colpus length (L), and distance between colpus (D) using ImageJ software. The pollen shape was determined by the P/E ratio and classified as follows: subprolate (1.14 < P/E < 1.33), prolate (1.33 ≤ P/E < 2.00), and perprolate (P/E > 2.00) [25].

### Pollen staining

Nine distinct staining protocols were employed to assess pollen viability, as detailed in Table 1. The experimental procedure was conducted as follows: 1 mL of the specific staining solution was pre-added to a 1.5 mL microcentrifuge tube. An appropriate amount of pollen was collected using a fine brush and gently tapped into the tube, followed by slight agitation to ensure a uniform suspension. After the precise reaction times specified in Table 1, approximately 30 μL of the reaction solution was pipetted onto the center of a glass slide and covered with a coverslip to prepare a temporary mount. The slides were immediately observed under a BX-53 optical microscope. For each staining treatment, three independent biological replicates were established. A total of ten representative fields of view were randomly captured across the replicates for each treatment (averaging approximately three fields per replicate). Within each field of view, at least 100 pollen grains were counted to determine the final pollen viability percentage.

**Table 1:** Methods to assess pollen viability.

Method	Staining solution and its preparation method	Staining time/(min)	Staining temperature/(℃)	Viable color or response
TTC [26]	3%TTC solution: Dissolve 0.6 g TTC powder and 12 g sucrose in 20 mL of distilled water.	30	35	Red
Carbol fuchsin [27]	Carbol fuchsin staining solution (Solarbio, Beijing, China)	5	25	Deep red or dark purplish-red
Acetocarmine [28]	1%Acetocarmine staining solution	5	25	Red
Methylene blue [29]	1% Methylene blue staining solution	10	25	Blue
Alexander [30]	10 mL 95% ethanol; 1 mL Malachite green (1% in 95% ethanol); 50 mL distilled water; 25 mL glycerol; 5 mL Acid fuchsin (1% aqueous solution); 0.5 mL Orange G (1% aqueous solution); 4 mL glacial acetic acid; finally, add 4.5 mL distilled water to reach a total volume of 100 mL.	10	25	Purple-red
Peroxidase [31]	Solution A: 0.5% Benzidine, 0.5% alpha naphthol, and 0.25% sodium carbonate in a 1:1:1 volume ratio. Solution B: 0.3% hydrogen peroxide (H_2_O_2_). Mix equal volumes of Solution A and Solution B during the test	30	35	Red
MTT [32]	0.05 g MTT dissolved in 10 mL Phosphate Buffered Saline	30	35	Dark purple
I_2_-KI [33]	1 g KI and 0.5 g I_2_ dissolved in 100 mL of distilled water	30	35	Blue
Red ink [24]	20% Red ink (Hero, Shanghai, China)	5	25	Red

### Pollen germination

Pollen *in vitro* germination was conducted using the solid culture method. The basal medium was prepared by dissolving 1 g of agar and 10 g of sucrose in 100 mL of distilled water. Single-factor experiments were performed by adding different concentrations of H_3_BO_3_, KNO_3_, KH_2_PO_4_, and Ca(NO_3_)_2_·4H_2_O to the basal medium, with the basal medium serving as the control (CK). Six concentration gradients were established for each factor as follows: H_3_BO_3_: 0.01, 0.05, 0.10, 0.20, 0.40, 0.80 g/L; KNO_3_: 0.001, 0.005, 0.010, 0.020, 0.040, 0.080 g/L; KH_2_PO_4_: 0.01, 0.04, 0.08, 0.10, 0.20, 0.40 g/L; Ca(NO_3_)_2_·4H_2_O: 0.01, 0.02, 0.04, 0.08, 0.12, 0.16 g/L. For each treatment, five independent biological replicates were prepared and cultured separately. Cultures were incubated in the dark at 30°C for 48 h. Subsequently, pollen germination was observed and photographed under an Olympus BX-53 microscope at 10 × magnification. Pollen germination was defined as the state where the pollen tube length exceeded the diameter of the grain. For each cultivated replicate, a representative field of view containing more than 100 pollen grains was randomly captured, and all grains within that field were counted to calculate the pollen germination rate. Nevertheless, both genotypes peaked at 0.10 g/L KH_2_PO_4_, where STZ-6 (41.53%) again surpassed STZ-9 (29.24%). Based on these single-factor results, the optimal concentration and its adjacent gradients for each element were selected to establish the factor levels for a subsequent L_9_(3^4^) orthogonal array optimization (Table 2).

**Table 2:** L_9_ (3^4^) is designed for an orthogonal experiment.

Treatment	Factor/(g/L)			
	H_3_BO_3_	KNO_3_	KH_2_PO_4_	Ca(NO_3_)_2_·4H_2_O
1	0.10	0.005	0.08	0.02
2	0.20	0.01	0.10	0.04
3	0.40	0.02	0.20	0.08

### Ethics approval and collection of plant materials

This species is widely distributed in the Guizhou Province, China, and all of which are cultivated varieties. *I. polycarpa* was collected from the *I. polycarpa* planting base in Guiding County, Guizhou Province, China. The collection site was tagged with GPS-GIS (longitude 107°25′03″E, latitude 226°59′01″N, elevation 1022 m), and prior written consent was obtained from the person in charge of the local nursery before collection. Specimens were identified by Chao Gao, a taxonomist from the Institute for Forest Resources and Environment of Guizhou, Guizhou University. Voucher specimens (Nos. TRXY25060820–TRXY25060821) were deposited in the Specimen Room of the Key Laboratory, College of Agro-forestry Engineering and Planning, Tongren University. *I polycarpa* is not listed in the International Union for Conservation of Nature (IUCN) Red List, with an assessment status of “Not Evaluated” (NE), indicating that the species has not been assessed on a global scale. In some local provinces (e.g., Guizhou Province), the species is categorized as “Least Concern”, reflecting its stable population status in this region. Thus, no specific permit was required for its collection. All procedures were conducted in compliance with institutional, national, and international guidelines for the ethical use of plant materials.

### Data analysis

Data were initially processed using Microsoft Excel 2019 (Microsoft Corporation, Redmond, WA, USA). Pollen morphological indices were measured using ImageJ software. One-way analysis of variance (ANOVA) and post-hoc multiple comparisons via Duncan’s multiple range test were performed at a significance level of α = 0.05 using SPSS 27.0 software (IBM Corp., Armonk, NY, USA). Line charts and box plots were generated using Origin 2024 (OriginLab Corporation, Northampton, MA, USA), and linear regression analysis, along with plotting, was conducted using R Studio 4.4.1 (Posit Software, PBC, Boston, MA, USA). Pollen germination rates and staining viability rates were determined based on three independent biological replicates and calculated using the following formulas


$$\begin{array}{c} Germination\;rate\;(\%)=\;\frac{Number\;of\;germinated\;pollen\;grains}{Total\;number\;of\;observed\;pollen\;grains}\times 100\% \end{array}$$

$$\begin{array}{c} Pollen\;staining\;viability\;rate\;(\%)=\;\frac{Number\;of\;viable\;pollen\;grains}{Total\;number\;of\;observed\;pollen\;grains}\times 100\% \end{array}$$

For the TTC, carbol fuchsin, acetocarmine, methylene blue, Alexander, peroxidase, MTT, and I_2_-KI staining protocols, pollen grains that exhibited distinct coloration were counted as viable. Conversely, for the red ink staining protocol, non-stained (colorless) pollen grains were classified as viable, while stained (red) grains were recorded as non-viable. All quantitative experimental data in the text, tables, and figures are expressed as Mean ± Standard Deviation (SD). A P-value < 0.05 was considered statistically significant.

## Results

### Pollen morphological characteristics

**Fig 2:**
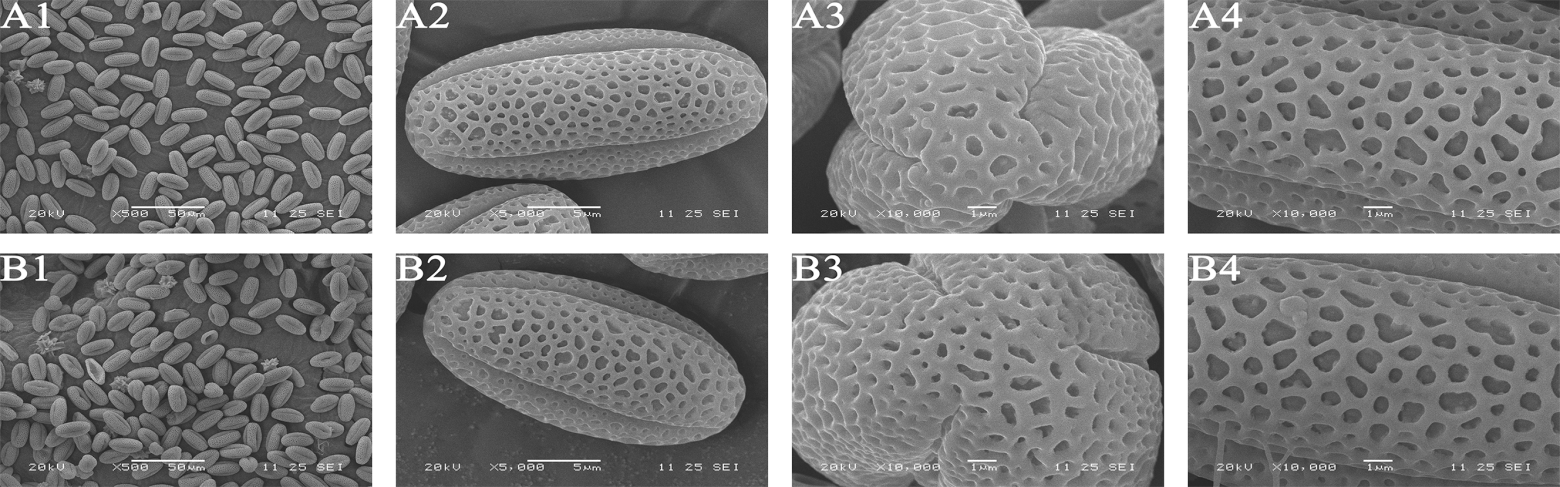
Pollen morphological characteristics of superior *I. polycarpa.* (A-B) represent pollen from STZ-6 and STZ-9, respectively. (1-4) indicate group view, equatorial view, polar view, and exine ornamentation of the pollen, respectively.

The pollen morphology of STZ-6 and STZ-9 displays a high degree of structural consistency (Fig 2). Both genotypes exhibit a 3-lobed circular shape in the polar view and an oblong shape in the equatorial view, classifying them as typical prolate pollen. Both are also characterized by tricolporate apertures and coarsely-reticulate exine ornamentation. Distinct variations in pollen dimensions were observed between the two elite genotypes. STZ-9 exhibited relatively larger pollen dimensions, with P and E values of 23.68 μm and 12.49 μm, respectively, while STZ-6 showed slightly smaller dimensions, with P and E values of 23.23 μm and 11.69 μm, respectively. The P/E ratio of STZ-6 (1.99) was higher than that of STZ-9 (1.90), suggesting that STZ-6 is morphologically more slender. The colpus length (L) of STZ-6 reached 19.53 μm, whereas STZ-9 had a longer colpus of 21.02 μm. Additionally, the distance between colpus (D) in STZ-9 (7.62 μm) was wider than in STZ-6 (7.22 μm). While the muri width was nearly identical between STZ-6 and STZ-9 (approximately 0.47–0.48 μm), the lumina diameter in STZ-9 (0.74 μm) was slightly larger than in STZ-6 (0.61 μm) (Table 3). In summary, observable morphological differences between STZ-6 and STZ-9 were found in specific indices, including colpus length, P/E ratio, and lumina size.

**Table 3:** Pollen morphological characteristics.

NO.	Polar Axis Length /μm	Equatorial Axis Length /μm	P/E Ratio	Colpus Length /μm	Colpus Distance /μm	Lumina Diameter /μm	Muri Width /μm	Polar View	Equatorial View	Exine Ornamentation	Colporate
STZ-6	23.23	11.69	1.99	19.53	7.22	0.61	0.48	Trilobate-circular	Prolate	Coarsely-reticulate	Tricolporate
STZ-9	23.68	12.49	1.90	21.02	7.62	0.74	0.47	Triloba-tecircular	Prolate	Coarsely-reticulate	Tricolporate

### Comparison of pollen staining methods

**Fig 3:**
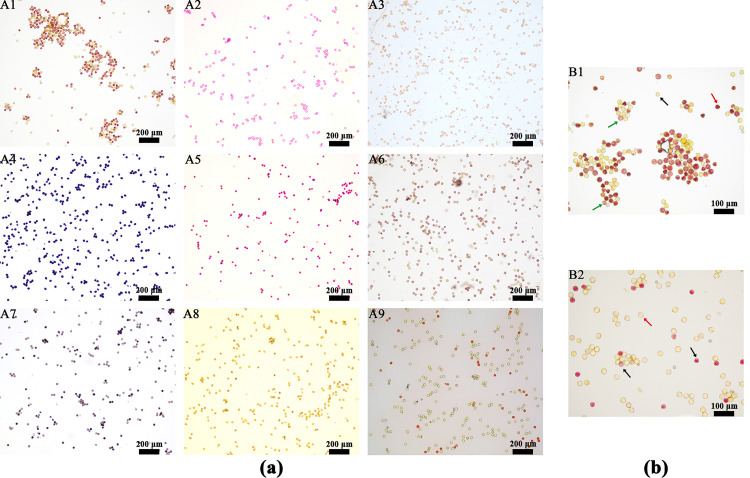
Evaluation of *I. polycarpa* pollen viability using different staining methods. (a) Comparison of nine staining techniques: (A1) TTC staining; (A2) Carbol fuchsin staining; (A3) Acetocarmine staining; (A4) Methylene blue staining; (A5) Alexander staining; (A6) Peroxidase staining; (A7) MTT staining; (A8) I_2_-KI staining; (A9) Red ink staining. All images were captured at ×10 magnification (scale bar = 200 μm). (b) Detailed comparison of effective staining methods: (B1) TTC staining, (B2) Red ink staining. Images were captured at ×20 magnification (scale bar = 100 μm). Red, green, and black arrows indicate viable, semi-viable, and non-viable pollen, respectively.

Distinct variations were observed in the effectiveness of the nine staining methods for *I. polycarpa* pollen (Fig 3). The I_2_-KI staining method failed to produce a distinct color reaction, with pollen remaining pale yellow or colorless, indicating that this method cannot detect viability through starch-specific reactions in this species. Staining protocols using carbol fuchsin, acetocarmine, methylene blue, and Alexander’s dye exhibited intense, non-specific global staining. Regardless of actual viability, nearly all pollen in the field of view was stained deep red, purple, or blue, making it impossible to distinguish between viable and non-viable pollen. Therefore, these methods are unsuitable for *I. polycarpa*. While the peroxidase and MTT staining methods did stain some pollen, the results were suboptimal. The MTT method resulted in dense, dark (black-purple) formazan crystals, which obscured the morphological features of the pollen and made it difficult to differentiate between viable and non-viable pollen under the microscope. Similarly, the peroxidase method suffered from high background interference and low differentiation. In contrast, the TTC and red ink staining methods provided the best differentiation. The TTC method specifically stains viable pollen red due to dehydrogenase activity, while non-viable pollen remains uncolored. The red ink method stained non-viable pollen red due to altered cell membrane permeability, while viable pollen excluded the dye, appearing colorless or pale yellow, thus creating a clear boundary between viable and non-viable pollen. In summary, the TTC and red ink methods were identified as the most effective for determining *I. polycarpa* pollen viability.

### Effects of individual components of medium on pollen germination rate of *I. polycarpa*

**Fig 4:**
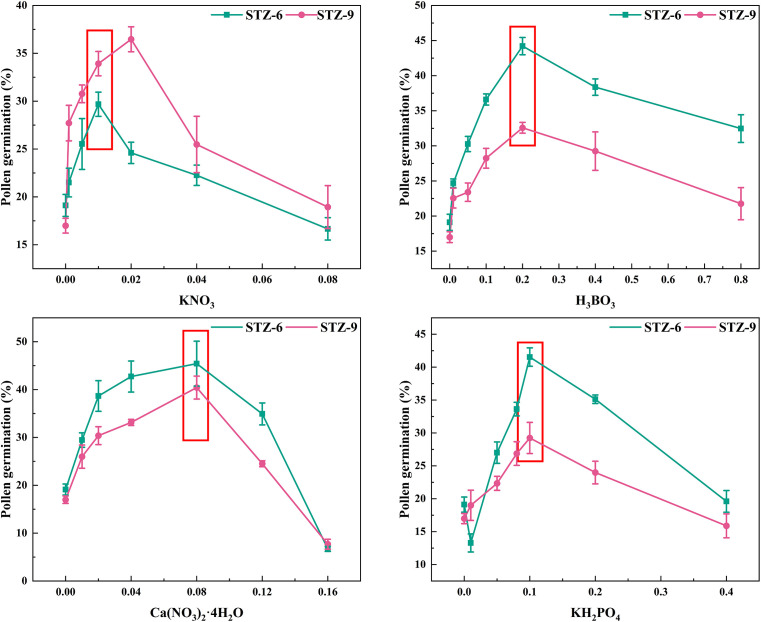
Pollen germination rates of *I. polycarpa* treated with varying concentrations of KNO_3_, H_3_BO_3_, Ca(NO_3_)_2_·4H_2_O, and KH_2_PO_4_.

The effects of the four mineral elements on the *in vitro* pollen germination of STZ-6 and STZ-9 exhibited distinct, element-specific regulatory patterns (Fig 4). Under H_3_BO_3_ and Ca(NO_3_)_2_·4H_2_O treatments, both genotypes displayed highly consistent trends. Specifically, when the H_3_BO_3_ concentration was 0.20 g/L, the germination rates of both STZ-6 and STZ-9 peaked at 44.21% and 32.57%, respectively. Among the tested elements, Ca(NO_3_)_2_·4H_2_O exerted the most pronounced promotive effect on germination, with both genotypes reaching their maximum germination rates at a concentration of 0.08 g/L. Notably, STZ-6 exhibited superior overall germination capacity compared to STZ-9. Under KNO_3_ treatment, however, STZ-9 outperformed STZ-6. At the optimal concentration of 0.010 g/L, STZ-9 had a germination rate of 36.47%, higher than STZ-6’s 29.68%, indicating a stronger response of STZ-9 to KNO_3_. In the KH_2_PO_4_ treatment, the responses of STZ-6 and STZ-9 to low concentrations were notably different. At 0.01 g/L KH_2_PO_4_, the germination rate of STZ-6 was lower than that of CK, suggesting high sensitivity to low-concentration ionic changes, which led to a transient inhibitory effect. Conversely, STZ-9 showed no such inhibition and displayed a continuous increase with rising concentrations.

### Optimization of *in vitro* germination medium for *I. polycarpa*

**Fig 5:**
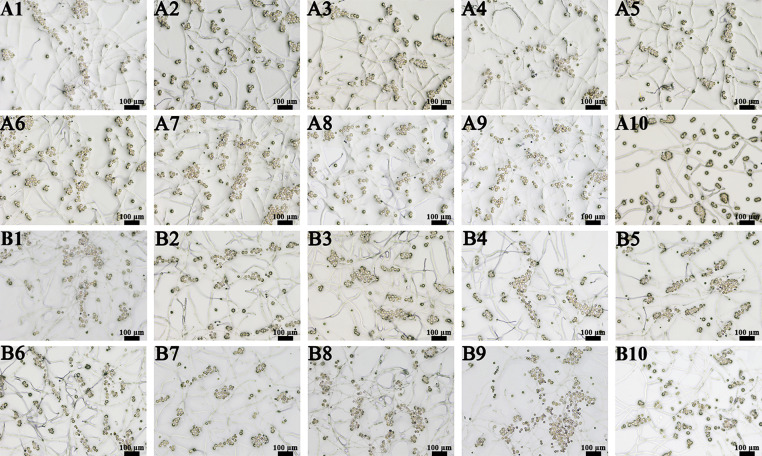
Effect of different medium formulations on the pollen germination rate of *I. polycarpa.* (A) and (B) represent genotypes STZ-6 and STZ-9, respectively. Numbers 1–9 correspond to mineral element combinations 1–9, and 10 represents the control (CK). Captured at ×10 magnification. Scale bar = 100 μm.

Results from the L_9_(3^4^) orthogonal experiment indicated that the concentration ratios of the tested mineral elements significantly affected the *in vitro* pollen germination rates of both genotypes (*P* < 0.05; Table 4 and Fig 5). For STZ-6 pollen, all treatment groups (No. 1 – No. 9) significantly promoted germination, yielding rates markedly higher than control (CK: 19.11%; *P* < 0.05). Among these, treatments No. 8. 6, and 9 exhibited the best effects, with germination rates of 48.48%, 47.55%, and 47.21%, respectively, showing no significant differences among them. Although no significant difference was observed between treatments No. 8 and No. 3, treatment No. 8 yielded the highest numerical value, representing a substantial increase of 153.69% compared to CK (19.11%). Consequently, treatment No. 8 was identified as the optimal medium formulation for STZ-6. Similarly, all treatment groups for STZ-9 exerted robust promotional effects; germination rates ranged from 38.53% to 48.47%, which more than doubled that of the control (CK: 16.99%). Treatment No. 6 performed best, reaching a peak of 48.47% (a 1.85-fold increase over CK), followed closely by treatment No. 4 (48.21%). Furthermore, treatment No. 6 was significantly superior to treatments No. 2, 3, and 5 (*P* < 0.05), thereby establishing it as the optimal formulation for STZ-9.

**Table 4:** Experimental results of *I. polycarpa* pollen germination based on L_9_(3^4^) orthogonal design.

No.	Mass concentration(g/L)				STZ-6	STZ-9
	H_3_BO_3_	KNO_3_	Ca(NO_3_)_2_·4H_2_O	KH_2_PO_4_	Germination rate/%	Germination rate/%
1	0.10	0.005	0.02	0.08	39.38 ± 2.00 de	46.59 ± 3.45 ab
2	0.10	0.01	0.04	0.10	41.59 ± 3.79 d	38.53 ± 3.55 d
3	0.10	0.02	0.08	0.20	46.02 ± 3.86 ab	42.46 ± 4.03 c
4	0.20	0.005	0.04	0.20	38.04 ± 2.68 e	48.21 ± 2.83 a
5	0.20	0.01	0.08	0.08	42.73 ± 1.80 c	44.02 ± 2.61 c
6	0.20	0.02	0.02	0.10	47.55 ± 5.42 a	48.47 ± 3.00 a
7	0.40	0.005	0.08	0.10	43.57 ± 2.61 bc	44.71 ± 3.27 bc
8	0.40	0.01	0.02	0.20	48.48 ± 2.02 a	45.29 ± 2.82 bc
9	0.40	0.02	0.04	0.08	47.21 ± 4.04 a	47.07 ± 2.86 ab
CK	0	0	0	0	19.11 ± 1.15 f	16.99 ± 0.77 e

Note: Different lowercase letters within the same column indicate significant differences at *P* < 0.05 according to Duncan’s multiple range test.

**Fig 6:**
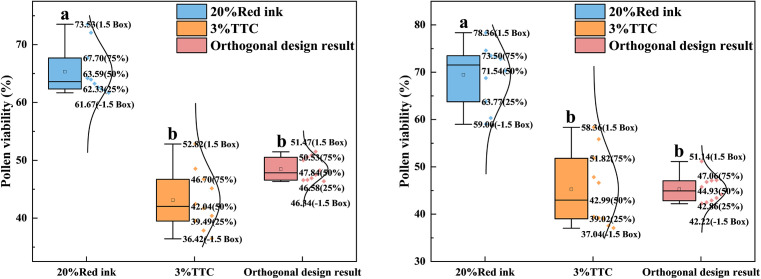
Comparison of pollen viability of STZ-6 and STZ-9 assessed by different methods. (a) STZ-6; (b) STZ-9. The box plots illustrate the distribution of pollen viability determined by red ink staining, TTC staining, and orthogonal experimental design results (*in vitro* germination). The horizontal line within the box represents the median, the box boundaries indicate the 25th and 75th percentiles, and the whiskers extend to 1.5 times the interquartile range. The scatter points represent individual data replicates, and the curved lines show the distribution density. Different lowercase letters (a, b) indicate significant differences at *P* < 0.05 according to *Duncan’s* multiple range test.

Further analysis revealed that compared to the optimized *in vitro* germination rates, the 20% red ink staining method distinctly overestimated pollen viability (Fig 6). Specifically, the red ink staining values for STZ-6 and STZ-9 reached 63.59% and 71.54%, respectively, falling within a high range of 60%–80%. These values were significantly greater than both the TTC staining results (42.04% and 42.99%) and the actual germination rates (47.84% and 44.93%) (*P* < 0.05). Although the viability rates determined by the 3% TTC method (42.04% for STZ-6; 42.99% for STZ-9) showed no statistically significant difference from the optimized germination rates (47.84% and 44.93%), the TTC dataset exhibited lower statistical stability than the orthogonal test outcomes. To eliminate such systematic biases between methods, the linear correlation between staining values and *in vitro* germination rates was further analyzed to construct a more accurate viability prediction model.

### Correlation analysis and accuracy evaluation of rapid staining protocols

**Fig 7:**
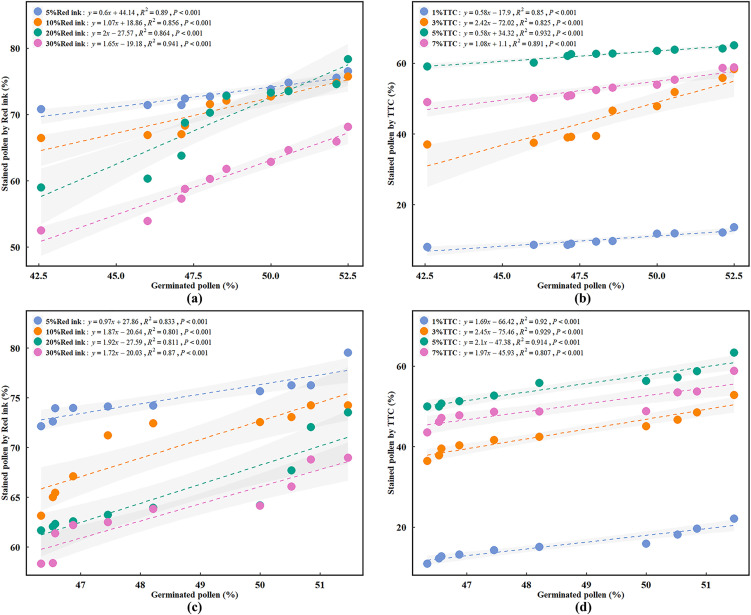
Single linear regression of *in vitro* germinated pollen (%) relative to stained pollen (%) for (a, c) red ink staining of STZ-9 and STZ-6, respectively. (b, d) TTC staining of STZ-9 and STZ-6, respectively.

To assess the accuracy of rapid staining protocols in predicting pollen viability, a simple linear regression model was established, using the *in vitro* germination values optimized through orthogonal experiments as the independent variable (X) and the staining results from various concentrations of red ink and TTC as the dependent variable (Y) (Fig 7). The results revealed a significant positive correlation between the staining values from both red ink and TTC methods and the orthogonal experimental design results. The red ink method showed overestimation in both STZ-6 and STZ-9, with viability values primarily ranging from 55% to 80%, consistently higher than the corresponding *in vitro* germination results on the X-axis. Linear regression analysis indicated that across different red ink concentrations, the R^2^ values ranged from 0.801 to 0.870 for STZ-6 and from 0.856 to 0.941 for STZ-9, demonstrating high goodness-of-fit (R^2^ > 0.80). The 30% red ink staining method yielded the highest R^2^ in both STZ-6 (R^2^ = 0.870) and STZ-9 (R^2^ = 0.941), exhibiting exceptional linear fitting accuracy and predictive stability. In contrast, the predictive performance of the TTC staining method displayed marked concentration dependence. The 1% TTC concentration significantly underestimated pollen viability, with its regression line falling far below the other groups (germination rate < 20%), despite maintaining R^2^ values of 0.850 and 0.920. As the TTC concentration increased to 5%, the staining measurements rose significantly and stabilized. Although the overestimation trend persisted, this concentration provided the optimal comprehensive linear fitting effect, with R^2^ values of 0.914 and 0.932, respectively. When the TTC concentration reached 7%, the measured viability showed a discernible decline compared to the 5% group, suggesting that excessively high TTC concentrations may inhibit pollen activity or the chromogenic reaction. Regression results confirmed that the 5% TTC method maintained excellent correlation for both samples, with R^2^ values of 0.914 (STZ-6) and 0.932 (STZ-9). In summary, the 30% red ink and 5% TTC treatments exhibited the highest goodness-of-fit and predictive reliability, making them the optimal staining protocols for rapid assessment of *I. polycarpa* pollen viability.

## Discussion

### Genetic conservatism and divergence of *I. polycarpa* pollen micromorphology

Pollen morphological characteristics are tightly controlled by genetic factors and exhibit high stability, making them essential for plant classification and germplasm identification [34,35]. SEM analysis revealed that the pollen of STZ-6 and STZ-9 displayed remarkable structural consistency. Both genotypes were prolate, tricolporate, and featured typical coarsely-reticulate exine ornamentation. These findings corroborate previous descriptions of *I. polycarpa* pollen morphology [36], while also showing distinct differences compared to other genera within the Salicaceae family. For example, Maciejewska et al. reported that pollen from certain Salicaceae genera is predominantly subprolate (62.7%) or prolate spheroidal (30.8%), with an average P/E ratio of 1.19, and is characterized by a reticulate pollen wall with lumina of varying sizes and often irregular outlines [37]. The present study confirms the genetic stability of morphological traits within the genus, reinforcing their role as a key basis for species classification and germplasm identification. Furthermore, research by El Ghazali, significant sexual diversity in monoecious species serves as a concrete manifestation of evolutionary effects [38]. Consistent with this, STZ-6 and STZ-9 displayed distinct micromorphological differences in quantitative pollen traits. Although both belong to the prolate category, the P/E value of STZ-6 (1.99) was higher than that of STZ-9 (1.90), suggesting a more slender and elongated shape. Notably, *I. polycarpa* pollen in this study exhibited dense lumina. It is hypothesized that the pollen surface is extensively covered by pollenkitt (composed primarily of lipids), enhancing pollen adhesion during entomophilous pollination [39]. This hypothesis warrants further exploration in future studies.

### Screening and evaluation of staining methods for *I. polycarpa* pollen

Accurate pollen viability assessment is critical for addressing challenges in hybrid breeding [40]. Staining assays are commonly used as indicators of viability due to their speed and simplicity compared to *in vitro* germination tests [41]. However, the effectiveness of a specific staining method varies significantly across plant species, primarily due to genetic differences in pollen constituents—such as starch, lipids, enzymes, and cell wall structures [42,43]. The results of this study indicate that the efficacy of conventional staining methods for *I. polycarpa* pollen is highly variable. The I_2_-KI staining method relies on a colorimetric reaction, where starch turns blue upon contact with iodine, signaling nutritional status and viability [44]. In this study, however, *I. polycarpa* pollen remained pale yellow or colorless after I_2_-KI treatment, failing to produce the characteristic blue color. This suggests that *I. polycarpa* pollen may be “lipid-rich” rather than “starch-rich”, rendering I_2_-KI ineffective—a phenomenon observed in other oil-bearing species such as *Camellia oleifera* [45] and *Elaeis guineensis* [46]. Moreover, morphological observations indicated that a dense pollenkitt layer on the surface of *I. polycarpa* pollen might further hinder the penetration of the I_2_-KI method. Structural stains, including carbol fuchsin, acetocarmine, methylene blue, and Alexander’s dye, exhibited non-specific, uniform coloration (false positives) across all pollen, making it impossible to distinguish viable from non-viable grains. This lack of differential staining has been widely reported in species such as *Arachis hypogaea* [47], *Olea europaea* [48], *Paeonia ostii* [49], and *Triticum aestivum* [50]. Although some studies have shown good applicability of acetocarmine in the genus *Thymus* [51] and high differentiation for Alexander’s, acetocarmine, and methylene blue in *Apium graveolens* [52], our findings highlight the significant species-specific variation of traditional structural staining methods. Enzymatic activity assays theoretically provide a more accurate reflection of the pollen’s physiological state; however, their applicability to *I. polycarpa* varies greatly. While the MTT method (based on dehydrogenase activity) colored some pollen, the resulting formazan crystals were densely deposited in *I. polycarpa* pollen cells. The dark black-purple coloration obscured morphological details, making it difficult to define the boundary between viable and non-viable pollen, leading to counting errors, consistent with prior studies [53]. Similarly, the peroxidase method was hindered by high background interference and low differentiation. In contrast, the TTC staining method (also based on dehydrogenase activity) exhibited better compatibility, producing a clear and vivid red reaction. Additionally, the red ink staining method, based on selective membrane permeability, demonstrated high differentiation. Consequently, this study identified the red ink and TTC staining methods as the optimal candidates for the rapid determination of *I. polycarpa* pollen viability, the accuracy of which was further validated by the subsequent *in vitro* germination experiment.

### Effects of mineral elements on pollen germination and optimization of *in vitro* culture conditions

The establishment of a high-quality *in vitro* germination system as a benchmark is essential for accurately assessing the reliability of candidate staining methods [54]. The components of the *in vitro* culture medium provide the necessary material for pollen tube growth and development. Single-factor and orthogonal experiments revealed that medium components significantly regulate *I. polycarpa* pollen germination. Boron is an essential micronutrient for plant growth, playing a critical role in fertilization, pollen germination, and pollen tube development, particularly in cell wall synthesis and structural maintenance [55]. Additionally, Ca^2+^ facilitates the regulation of polar growth and orientation in the pollen tube [56]. The results indicated that both H_3_BO_3_ and Ca(NO_3_)_2_·4H_2_O, at appropriate concentrations, significantly enhanced pollen germination rates in both STZ-6 and STZ-9. These findings align with previous research showing that optimal concentrations of boron and calcium promote reproductive growth in plants, consistent with reports for other economic tree species such as *Pyrus pyrifolia* [57] and *Paeonia ostii* [49], thereby confirming the universal importance of boron and calcium in pollen development. However, potassium (K) and phosphorus (P) requirements exhibited significant genotypic differences between the two elite *I. polycarpa* genotypes. STZ-9 showed a more sensitive response to KNO_3_, which may be attributed to variations in potassium ion channel activity and osmotic regulation across different pollen genotypes. Conversely, STZ-6 showed sensitivity and inhibition under low-phosphorus conditions, possibly due to differences in its energy metabolism, though the specific mechanisms remain unclear and require further investigation. Orthogonal optimization identified distinct optimal media for the two genotypes: for STZ-6, the optimal medium consisted of 0.40 g/L H_3_BO_3_ + 0.01 g/L KNO_3_ + 0.02 g/L Ca(NO_3_)_2_·4H_2_O + 0.20 g/L KH_2_PO_4_; for STZ-9, the optimal medium comprised 0.20 g/L H_3_BO_3_ + 0.02 g/L KNO_3_ + 0.02 g/L Ca(NO_3_)_2_·4H_2_O + 0.10 g/L KH_2_PO_4_. These discrepancies likely reflect genotypic variations between the elite trees [58]. Nevertheless, both media improved the overall germination rates by 1.5 to 1.8 times compared to the control group. This optimized *in vitro* germination system accurately represents the genuine potential of pollen to complete fertilization, providing a reliable baseline for subsequent evaluations of staining method accuracy.

### Discrepancy analysis between stained values and actual germination rates with methodological recommendations

By comparing the selected staining methods with the optimized *in vitro* germination results, a critical issue was identified: the red ink staining method significantly overestimated pollen viability, yielding values considerably higher than the actual germination rates (*P* < 0.05), while the TTC measurements showed no significant difference from the actual germination rates, though slightly lower. This discrepancy highlights the inherent limitations of staining methods in reflecting the full complexity of pollen physiology. The red ink method, in particular, produced the largest overestimation error (approximately 1.6-fold in STZ-6). This overestimation can be attributed to the progressive nature of pollen senescence; even when pollen has lost its metabolic functionality, it may still retain relatively intact cell membranes that exclude the dye, leading to their misidentification as viable [59]. Detection methods that rely solely on structural integrity fail to exclude pollen that, while structurally intact, has lost the biological capacity to germinate, thereby resulting in a high false-positive rate. In contrast, although the TTC method yielded results closer to actual germination rates, these values still slightly lower. The TTC method, which relies on dehydrogenase activity to produce coloration, reflects the intensity of respiratory metabolism [60]. However, enzymatic activity alone does not imply full germination potential. Pollen germination is a complex physiological process involving organelle movement, cytoplasmic streaming, and vesicle transport, all of which are crucial for sustaining pollen tube growth [61]. Even if residual dehydrogenase activity allows for TTC staining, the pollen remains biologically “non-functional” if its nucleus has degraded or if it lacks sufficient energy reserves to support sustained pollen tube elongation. Therefore, TTC staining values represent the “germination potential” rather than the realized ability of pollen to germinate. In conclusion, while the *in vitro* germination method is more time-consuming, it provides a direct and accurate reflection of the true potential of *I. polycarpa* pollen to complete fertilization, making it the most reliable benchmark for assessing pollen viability.

### Correction of staining results based on linear regression models and hierarchical application

To address the limitations of staining methods, which are operationally convenient but exhibit physiological deviations, and the cumbersome procedures of *in vitro* germination, this study developed a correction system for staining results and proposed a hierarchical application strategy based on orthogonal experimental data. By establishing simple linear regression models, this study not only elucidates the conversion relationship between staining values and actual germination potential in *I. polycarpa* but also provides a scientific framework for selecting methods across different application contexts. The results indicate that although red ink staining tends to overestimate pollen viability, its *R*^2^ with the orthogonal experimental results remains consistently above 0.800. This suggests that the error associated with this method represents a stable, systematic bias, with the 30% red ink staining yielding the most reliable predictive performance (*R*^2^ values of 0.870 and 0.941). The red ink method, relying on simple physical osmosis, generates coloration within minutes and requires minimal ambient temperature control or specialized equipment. Characterized by high timeliness and operational portability, red ink staining is especially suited for preliminary field screening of pollen quality. Before field pollination, precise germination data may not be necessary; instead, using the established regression equations, pollen usability can be rapidly assessed by correcting the red ink viability values. In contrast, the TTC method demands more stringent detection conditions and exhibits notable concentration dependence. At a low concentrations (1%), TTC produces false negatives due to insufficient substrate, whereas a 5% TTC concentration, under substrate-saturated conditions, achieves strong consistency with the actual germination rate (*R*^2^ values of 0.914 and 0.932). Consequently, for scientific research requiring high accuracy, such as the selection of hybrid parents for *I. polycarpa*, the 5% TTC method, coupled with the linear correction model, proves to be the optimal solution. This approach bypasses the time-consuming *in vitro* germination process while closely approximating actual physiological activity, thus balancing both efficiency and accuracy. In summary, raw readings from staining methods often overestimate viability, failing to reflect true pollen germination potential. By accounting for the overestimation bias inherent in staining methods, this study proposes an evaluation framework combining “Preliminary Field Testing with Red Ink and Precise Laboratory Testing with TTC.” This strategy provides a scientific basis for high-efficiency artificial pollination in *I. polycarpa.*

### Limitations and future perspectives

Although this study successfully constructed a high-efficiency staining correction system and a hierarchical application framework for *I. polycarpa*, several inherent limitations should be acknowledged to guide future research. First, while we optimized media components and cross-validated viability methods at specific sampling nodes, the temporal biological characteristics of pollen remain unmapped. Furthermore, factors such as flowering phenology [62], daily pollen shedding rhythms [63], the exact window of peak viability [64], and post-harvest pollen longevity [65] are well-known to critically influence pollen quality. Identifying these optimal collection windows is an essential step for maximizing the real-world efficiency of breeding programs. Second, while our experimental materials were focused on two elite male genotypes (STZ-6 and STZ-9), the empirical formulations and regression equations derived herein are intended to serve as a practical reference standard and methodological baseline for *I. polycarpa*. Although pollen morphology and nutrient responsiveness exhibit genotypic variations, these specific elite genotypes provide high-value benchmarking data. Consequently, this framework offers a foundational platform that can be extended to a broader range of male individuals from diverse natural populations in future scaling-up studies.

Similarly, how this *in vitro* system translates into field operations remains to be validated. While it provides a precise laboratory benchmark, its direct utility in large-scale artificial pollination, pollen quality screening, pollinizer selection, storage management, and fruit-set prediction requires systematic testing. Establishing a direct correlation between corrected pollen viability and final fruit-set rates through field trials was not performed in this staged laboratory work, but it remains a primary focus for our upcoming research. In conclusion, the “Red Ink-TTC” combination approach provides a practical baseline for evaluating pollen quality. By progressively integrating phenological data, broader genetic resources, and field-scale verifications, this protocol can be effectively refined to meet the demands of commercial *I. polycarpa* production.

## Conclusions

*I. polycarpa* pollen exhibits genetic conservatism at the genus level, consistently characterized by prolate shapes, tricolporate apertures, and coarsely-reticulate exine ornamentation, while showing notable micromorphological variation among intra-specific genotypes. Among the nine evaluated staining methods (TTC, carbol fuchsin, acetocarmine, methylene blue, Alexander, peroxidase, MTT, I_2_-KI, and red ink), TTC and red ink emerged as the most effective, providing clear chromatic differentiation to distinguish viable from non-viable pollen. *In vitro* germination assays demonstrated that optimal concentrations of H_3_BO_3_, KNO_3_, Ca(NO_3_)_2_·4H_2_O, and KH_2_PO_4_ significantly enhanced pollen germination. Orthogonal optimization identified the ideal medium for genotype STZ-6 as 0.40 g/L H_3_BO_3_ + 0.01 g/L KNO_3_ + 0.02 g/L Ca(NO_3_)_2_·4H_2_O + 0.20 g/L KH_2_PO_4_, while for STZ-9, the composition was 0.20 g/L H_3_BO_3_ + 0.02 g/L KNO_3_ + 0.02 g/L Ca(NO_3_)_2_·4H_2_O + 0.10 g/L KH_2_PO_4_. Additionally, a significant linear correlation between staining values and germination rates led to the development of a hierarchical evaluation strategy: the 5% TTC method is recommended for precise laboratory quantification, while the 30% red ink method is suitable for rapid, field-based pollen quality screening. These findings provide a standardized framework for evaluating *I. polycarpa* pollen viability and establish a strong scientific foundation for future research on its reproductive biology, as well as the optimization of artificial pollination and breeding programs.
